# Political economy analysis for health

**DOI:** 10.2471/BLT.19.238311

**Published:** 2019-08-01

**Authors:** Michael R Reich

**Affiliations:** a Harvard T.H. Chan School of Public Health, Harvard University, 677 Huntington Ave, Boston, MA 02115, United States of America.

Achieving the health targets of the sustainable development goals (SDGs) depends on favourable political economy factors. Policy reform can be best understood, analysed and managed by recognizing how political economy shapes what happens in the reform process. Political economy can be broadly defined as the study of both politics and economics, specifically the interactions between them and their consequences for specific outcomes of interest.^1^

Political economy focuses on power and resources, how they are distributed and contested in different country and sector contexts, and the resulting implications for development outcomes. Applied political economy analysis supports policy processes in three ways. First, such analysis generates an assessment of the political landscape, including a stakeholder map, an assessment of power and position of key political actors, and an estimate of political feasibility for policy change. Second, the analysis focuses attention on how political strategies shape the feasibility of a policy reform and on the importance of politically managing the change process. Third, such analysis underlines the role of political economy factors throughout the policy cycle, including agenda-setting, policy design, adoption, implementation and evaluation.

The health sector can make better use of knowledge about politics, power and political analysis to improve the effectiveness of its policy process. Health policy-makers recognize the need for political economy analysis; however, they often lack a clear idea of how to do it. The World Health Organization (WHO) can promote evidence-based political economy analysis to assist health policy-makers in managing change more effectively, for instance to move towards universal health coverage (UHC) or to advance other health-related SDGs.

WHO is increasingly engaged in political economy analysis that is incorporated into technical guidance. For example, WHO’s toolkit to implement Article 5.2(a) of the Framework Convention on Tobacco Control includes political analysis in parallel with technical analysis to strengthen national coordinating mechanisms for tobacco control.^2^ Furthermore, the programme of work on the Political Economy of Health Financing Reform^3^ has sponsored a series of papers, including an overview paper plus country studies and how-to guides, has been published in a special issue of *Health systems & reform*.^4^ Last, the Global Malaria Programme has developed methods for the assessment of political commitment for malaria elimination at the national level.^5^

These activities, as well as others, reflect efforts throughout the development community to incorporate political economy analysis. Involved actors include the United Kingdom Department for International Development,^6^ the World Bank,^7^ the Swedish International Development Agency, the Australian Agency for International Development and the United States Agency for International Development.^8^ A website entitled “Thinking and working politically: community of practice”, provides publications on political economy for both practitioners and academics.^9^ Political analysis is also integrated into training and practice on health reform.^10^ In January 2019, the Prince Mahidol Award Conference examined the political economy of noncommunicable diseases, the first major global health conference to include political economy in its title and as its focus.^11^

One core challenge for political economy proponents is to provide a robust method of analysis that is easily learned and applied by practitioners to generate usable knowledge and assist in policy decisions and effective implementation. Various how-to do guides exist on political economy analysis for development,^1^^,^^6^^,^^7^ but few assessments have been conducted. A freely available software package for applied political analysis, PolicyMaker 4.0,^12^ combines stakeholder analysis with political strategy development in a Windows-based software and has been used in many countries around the world. 

WHO can advance political economy analysis in health through specific actions. The organization could give systematic attention to political economy factors and analysis, building on a series of papers organized by WHO on the political economy of health financing reform.^4^ WHO could hire more staff with training and experience in applied political economy. As well, the organization could give practical guidance to national health ministries on how to do applied political economy analysis and how to effectively incorporate this evidence into efforts to improve public health. These three steps would help WHO engage more effectively with political economy as part of the organization’s overall technical support, to advance health goals at both the global and the national levels.
